# The relative contributions of visual and semantic information in the neural representation of object categories

**DOI:** 10.1002/brb3.1373

**Published:** 2019-09-27

**Authors:** Lindsay W. Victoria, John A. Pyles, Michael J. Tarr

**Affiliations:** ^1^ Department of Psychology, The Center for the Neural Basis of Cognition Carnegie Mellon University Pittsburgh Pennsylvania

**Keywords:** functional magnetic resonance imaging, semantics, visual cortex, visual perception

## Abstract

**Introduction:**

How do multiple sources of information interact to form mental representations of object categories? It is commonly held that object categories reflect the integration of perceptual features and semantic/knowledge‐based features. To explore the relative contributions of these two sources of information, we used functional magnetic resonance imaging (fMRI) to identify regions involved in the representation object categories with shared visual and/or semantic features.

**Methods:**

Participants (*N* = 20) viewed a series of objects that varied in their degree of visual and semantic overlap in the MRI scanner. We used a blocked adaptation design to identify sensitivity to visual and semantic features in a priori visual processing regions and in a distributed network of object processing regions with an exploratory whole‐brain analysis.

**Results:**

Somewhat surprisingly, within higher‐order visual processing regions—specifically lateral occipital cortex (LOC)—we did not obtain any difference in neural adaptation for shared visual versus semantic category membership. More broadly, both visual and semantic information affected a distributed network of independently identified category‐selective regions. Adaptation was seen a whole‐brain network of processing regions in response to visual similarity and semantic similarity; specifically, the angular gyrus (AnG) adapted to visual similarity and the dorsomedial prefrontal cortex (DMPFC) adapted to both visual and semantic similarity.

**Conclusions:**

Our findings suggest that perceptual features help organize mental categories throughout the object processing hierarchy. Most notably, visual similarity also influenced adaptation in nonvisual brain regions (i.e., AnG and DMPFC). We conclude that category‐relevant visual features are maintained in higher‐order conceptual representations and visual information plays an important role in both the acquisition and neural representation of conceptual object categories.

## 1 INTRODUCTION

Object categories form the core of how we think and reason about the world, and understanding the neural basis of object category representations is fundamental to the study of human cognition. One thread running through this domain of research is consistent disagreement over the degree to which mental categories are formed on the basis of visual features versus semantic features (Keil, Smith, Simons, & Levin, 1998; Sloutsky, 2010). A critical challenge in empirically addressing this question is dissociating the relative contributions of visual and semantic sources of information.

This study focuses on how the brain integrates visual information (i.e., higher‐order processing of perceptual features and unified object form) and semantic information (i.e., conceptual knowledge of how objects are associated with each other) to define and maintain object categories. Both sources of information play a combined role in the representation of object category boundaries and are typically correlated, as function closely follows form (e.g., birds have wings, and tools have handles; Tang et al., 2018). However, the organization of visual and semantic representations within the broad network of category‐selective brain regions remains unspecified.

Our decision to interrogate category‐selective brain regions is based on previous research that implicates such areas as important in the processing of object categories. With respect to object categories, visual features are processed and encoded along a neural pathway that begins in early visual cortex and extends dorsally and ventrally through the occipital and temporal lobes (Freud, Culham, Plaut, & Behrmann, 2017; Ishai, Ungerleider, Martin, Schouten, & Haxby, 1999). This visual pathway is organized in a hierarchical fashion, with basic pixel‐level visual features (e.g., edges, contrast, size) being processed in early visual cortex and increasingly higher‐order perceptual features (e.g., unified global shape, animacy, category membership) being processed more anteriorly along the dorsal and ventral visual cortex (Grill‐Spector & Malach, 2001; Grill‐Spector & Weiner, 2014).

It is well established that the ventral visual cortex is critical for higher‐order object processing and unified object representations. Within this ventral “stream,” the lateral occipital cortex (LOC) is the neural substrate most often associated with *visual category representations* (Coggan, Liu, Baker, & Andrews, 2016; Kravitz, Saleem, Baker, Ungerleider, & Mishkin, 2013; Malach et al., 1995). Here, we focused on the LOC as an a priori region of interest because of its role in integrating individual features into coherent perceptions representations of objects at a global level, which may be particularly relevant for defining associations among object category members at the visual level. A functional role for the LOC in object perception is supported by the presence of an adaptation response that presents as a decrease in neural activity across the LOC in conjunction with repeated viewings of identical stimuli or visually similar stimuli (Kim, Biederman, Lescroart & Hayworth, 2009; Sayres & Grill‐Spector, 2006). This pattern of attenuated activation within LOC is assumed to occur in response to shared visual features at some intermediate or high levels of visual representation.

To the extent that different object categories are based on different sets of informative features (in other words, the features that provide the most relevant information in defining complex associations among object category members; e.g., balls are round and roll, sweaters have sleeves and cover our arms), distinct functional neural circuits supporting the processing of each particular feature domain will become associated with categories (Bi, Wang, & Caramazza, 2016; Mahon & Caramazza, 2011). Therefore, *semantic category representations*—assumed to incorporate conceptual, knowledge‐based features—are likely to be associated with a wider network of regions distributed across the brain (Huth, Nishimoto, Vu, & Gallant, 2012; Martin, 2007; Ralph, Jefferies, Patterson, & Rogers, 2017).

With respect to evidence for a distributed semantic network, regions of the prefrontal cortex are often implicated in semantic processing tasks. The prefrontal cortex is associated with memory processes, the hypothesis being that they play an important role in the acquisition, storage, and retrieval of knowledge that forms the conceptual basis of object categories (Martin & Chao, 2001; Wagner, Koutstaal, Maril, Schacter, & Buckner, 2000). More precisely, the prefrontal cortex may support the maintenance of conceptual category boundaries in working memory, contributing to goal‐directed behavior and efficient processing of task‐relevant stimulus dimensions (Lee & Baker, 2016). Other key brain areas that appear to be recruited for semantic category representation include motor regions, particularly precentral gyrus (PrG), which closely borders premotor planning regions and integrates motor information for the functional‐based processing of objects being used in an action context (Liljeström et al., 2008; Martínez et al., 2014). These regions are crucial for the maintenance of conceptual object category boundaries arising from common functional properties among associated objects, and, as such, enable efficient interaction with novel objects and the application of similar actions to all objects from within the same category (Matheson, Buxbaum, & Thompson‐Schill, 2017).

To further our understanding of the neural representation of higher‐order semantic features, we chose to focus on a superordinate object category distinction that has been found to elicit neural activation in category‐selective regions widely across the brain: living versus nonliving objects. While this distinction has been widely studied in the past (e.g., Caramazza & Mahon, 2003; Fuggetta, Rizzo, Pobric, Lavidor, & Walsh, 2008; Martin & Chao, 2001; McRae, Cree, Seidenberg, & McNorgan, 2005), and it is only one of many possible superordinate category distinctions we could have focused on, the difference between living and nonliving objects is particularly relevant to the present research. Most saliently, the inclusion of living and nonliving objects in the present study will allow us to examine the role of perceptual similarity in determining conceptual object category boundaries. While living versus nonliving categories are associated with distinct neural substrates, previous research indicates that increased perceptual similarity and statistical regularities among living objects may drive this neural division (Farah & McClelland, 1991; Sadeghi, McClelland, & Hoffman, 2015; Torralba & Oliva, 2003). By controlling for the extent to which visual features are shared (or not shared) among our living versus nonliving category members, we may also determine the extent to which this conceptual category boundary is defined by visual feature representations in the brain.

We aimed to investigate the degree of overlap versus the distinctiveness of the neural representations of visual and semantic information by systematically manipulating both sources of information within our object stimulus set. In theory, one could design novel visual stimuli that dissociate perceptual features from semantic features, crossing visual similarity (or dissimilarity) with semantic category overlap (or lack thereof). In practice, this dissociation is difficult to realize; novel objects that look similar are typically treated, by default, as members of the same semantic category (Farhadi, Endres, Hoiem, & Forsyth, 2009; Frome et al., 2013; Landau, Smith, & Jones, 1988; O'Reilly, Wyatte, Herd, Mingus, & Jilk, 2013). Therefore, we adopted a design in which we identified familiar, everyday objects that were *similar or dissimilar in shape*, but belonged to the *same semantic category*, toward the goal of better understanding the contribution of visual information to the formation and organization of semantic object categories. This design allowed us to examine the extent to which regions throughout the brain are sensitive to differing combinations of both visual and semantic features. Inherent in adopting this design was our difficulty in satisfactorily identifying objects that were similar in shape but belonged to *different semantic categories*, as similarly shaped objects cannot help but share at least some semantic features.

Two recent papers adopted a similar approach but included a condition of perceptual similarity crossed with semantic dissimilarity (Bracci & de Beeck, 2016; Martin, Douglas, Newsome, Man, & Barense, 2018). Prima facie, both of these studies managed to overcome our concerns with this condition. However, we suggest that while nominally semantically dissimilar, perceptually similar objects continue to share many functional semantic features. Martin et al. (2018) posit that hairdryers, electric drills, and handguns share similar shapes, but are conceptually distinct. However, these objects have many semantic properties in common: they are graspable, have handles, and are commonly held in a similar orientation, etc. Bracci and de Beeck (2016) likewise include a condition in which perceptually overlapping shapes nominally differ in conceptual category. Again, these nominally semantically dissimilar objects still share functionally based semantic properties (e.g., a paintbrush and a ping pong paddle are both gripped by their handles).

In light of the complexities and potential confounds inherent in a similar shape/ different categories condition, we chose to control our stimulus manipulations as much as possible and focus our study exclusively on familiar, object categories comprised of semantically related exemplars with overlapping or nonoverlapping visual shapes; thus, our object categories shared the same degree of semantic similarity but varied in their degree of visual similarity. This design was used in conjunction with functional magnetic resonance imaging (fMRI) to investigate whether category‐selective brain regions are sensitive to the following: (a) semantic overlap irrespective of visual similarity, (b) visual overlap irrespective of semantic similarity, or (c) are not differentially sensitive to visual versus semantic overlap and thus process a mix of visual and semantic features. Therefore, to investigate the degree to which shared visual features contribute to category selectivity across the broad set of brain regions associated with category representation, we varied the degree of *visual feature overlap* among category members while holding semantic category membership consistent across blocks of objects, thereby allowing us to assess the contribution of perceptual similarity in defining category‐selective neural representations. We used a whole‐brain analysis approach to identify a network of regions that are critical for the maintenance of semantic category boundaries in the absence of visual similarity. The inclusion of objects from the same semantic categories that were either visually similar or dissimilar allowed us to separate those brain regions sensitive to shared perceptual features from those regions sensitive purely to semantic information.

We predicted we would observe neural adaptation in response to visual similarity throughout the visual processing hierarchy, beginning in early visual cortex, and extending into the ventral visual pathway. LOC was expected to demonstrate greater adaptation for visually similar categories with higher degree of perceptual feature overlap and to exhibit less sensitivity semantic information, with little difference in adaptation between living and nonliving category boundaries. Further, we predicted that a wider network of processing regions extending in an anterior direction in the brain would demonstrate adaptation in response to conceptual category boundaries processing in the absence of visual similarity. Specifically, greater adaptation was expected in prefrontal and premotor regions for categories based predominantly on semantic features (e.g., living vs. nonliving). To the extent that these regions are sensitive to semantic information, differential adaptation effects for visually similar and dissimilar category members were not expected.

## 2 MATERIALS AND METHODS

### 2.1 Participants

Participants were 20 undergraduate and graduate students from Carnegie Mellon University, aged 18–30 (*M* = 21.6 years). Participants were right‐handed native English speakers with normal or corrected‐to‐normal vision. Participants gave written informed consent as approved by the Carnegie Mellon University Institutional Review Board and received monetary compensation for their participation.

### 2.2 Stimuli

Stimuli consisted of 496 images of single objects. Objects and corresponding images were initially selected by the experimenters and were behaviorally piloted in a picture‐naming task to confirm that participants assigned correct object names (e.g., the word “apple” to a picture of an apple) with 98% accuracy. Two hundred eighty of the objects were visually similar or dissimilar objects grouped into subsets drawn from the same semantic categories. Stimuli were grouped in subsets of ten objects: 16 groups were semantically associated objects with a high degree of visual feature overlap between objects, and 16 groups were semantically associated objects that were visually distinct from one another. An additional 16 objects were used in blocks comprised of identical repetitions, and 160 visually and semantically unrelated objects were used in blocks comprised of random objects (i.e., a mix of living and nonliving objects with no repetitions and no overlap with objects in semantically associated categories). The identical and random conditions were included as they were expected to elicit maximum adaptation effects (due to entirely overlapping visual and semantic features for identical objects) and minimum adaptation effects (due to nonoverlapping visual and semantic features for random objects).

**Figure 1:**
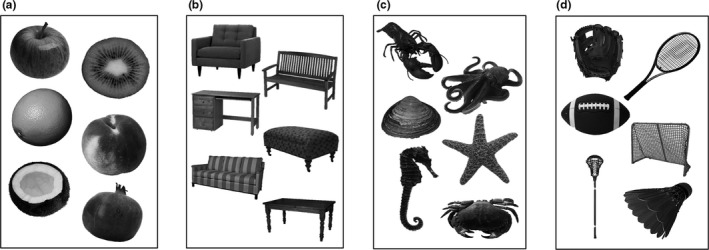
Examples of the stimuli from each block condition in the fMRI paradigm. (a) Visually similar objects from the same living category. (b) Visually similar objects from the same nonliving category. (c) Visually dissimilar objects from the same living category. (d) Visually distinct objects from the same nonliving category

The visually similar and dissimilar groups were further divided into eight groups of living objects and eight groups of nonliving objects (Figure 1). Table 1 lists all object categories for the visually similar versus dissimilar conditions and the living versus nonliving conditions.

**Table 1:** List of object categories included in fMRI‐adaptation paradigm as a function of visual similarity and living/ nonliving category membership

	Living	Nonliving
Visually similar	Animals (Furry)	Clothes
	Animals (Hooved)	Food/Snacks
	Birds	Furniture
	Fish/Ocean	Instruments
	Flowers	Office Supplies
	Fruits	Shoes
	Reptiles	Sports
	Vegetables	Vehicles
Visually dissimilar	Animals (Jungle)	Clothes
	Animals (Forrest)	Food/Snacks
	Birds	Instruments
	Fish/Ocean	Kitchen Gadgets
	Fruits	Office Supplies
	Insects	Sports
	House plants	Tools
	Vegetables	Vehicles

We quantified perceptual similarity in the visually similar and dissimilar object categories with a Gabor filter analysis, which measured the overlap in frequency for our stimulus images on a pixel‐by‐pixel level. We used the Gabor filter approach because it is a mathematically useful way of quantifying spatial frequency information and it considers multiple levels of low‐ and high‐level image features that were of interest in our study, including image texture (Idrissa & Acheroy, 2002), orientation (Kong, 2008; Sagi, 1990), and edge detection (Jiang, Lam, & Shen, 2009; Mehrotra, Namuduri, & Ranganathan, 1992).

The mean Gabor distance between all objects in each object group was analyzed as a function of condition. Visually similar objects were closer in Gabor distance (*M* = 0.31, *SD = *0.03) than visually dissimilar objects (*M* = 0.41, *SD = *0.03) and random objects (*M* = 0.49, *SD = *0.04). The difference in Gabor distance was significant for similar versus dissimilar objects (*t*(15) = −3.37, *p* < .005) and similar versus random objects (*t*(15) = −5.80, *p* < .001), but there was no significant difference between visually dissimilar objects and random objects (*t*(15) = −1.93, *p* = .07). Further, the Gabor distance between living objects (*M* = 0.34, *SD = *0.02) and nonliving objects (*M* = 0.39, *SD* = 0.03) was not significant (*t*(15) = −1.41, *p* = .18).

To control for semantic similarity across all conditions, word association norms were calculated for all object groups (USF Free Association Norms; Nelson, McEvoy, & Schreiber, 1998). Semantic similarity is defined as participants’ preexisting knowledge of shared conceptual category membership among stimulus objects (e.g., balls, bats, and gloves are used to play baseball; guitars, drums, and trumpets are used to make music). This construct was quantified by a word association norm score representing the likelihood of one object label calling to mind another object label (Nelson, McEvoy, & Dennis, 2000; Nelson, McEvoy, & Schreiber, 2004). Norms were calculated for both within‐category associations (e.g., *apple* to *cherry* and *orange*) and higher‐order category member associations (e.g., *apple* to *fruit* and *chair* to *furniture*). This analysis indicated that visually similar (*M* = 0.09, *SD = *0.07) and dissimilar objects (*M* = 0.10, *SD = *0.05) did not differ from one another in semantic relatedness for within‐category associations (*t*(27) = −0.45, *p* = .65). There was a marginally significant difference in category member associations, with visually similar (*M* = 0.22, *SD = *0.18) objects being more associated than dissimilar objects (*M* = 0.13, *SD = *0.07) with their higher‐order category label (*t*(29) = 1.96, *p* = .06). While we acknowledge that this trend toward a difference in category membership as a function of visual similarity may have some role in defining category boundaries, we do not expect it to impact the results of this study as we are primarily focused on within‐category representations of individual objects, as opposed to higher‐to‐lower order linkages. Finally, semantic relatedness did not differ as a function of living versus nonliving category membership for the within‐category associations (Living: *M* = 0.09, *SD* = 0.06; Nonliving: *M = *0.10, *SD = *0.06, *t*(27) = −0.56, *p* = .58) or the category member associations (Living: *M* = 0.21, *SD* = 0.16; Nonliving: *M = *0.14, *SD = *0.11, *t*(29) = 1.40, *p* = .17). We did not examine semantic similarity among objects in the identical and random control conditions because these conditions were not included in the analysis of differences in adaptation across object categories within semantic processing regions.

### 2.3 Procedure

#### 2.3.1 fMRI‐adaptation paradigm

We adopted a block adaptation design to identify those brain regions associated with the processing of specific stimulus attributes (Grill‐Spector & Malach, 2001). The canonical signature of an adaptation design is a reduction in neural signal upon repeated viewings of images overlapping along some low‐ or high‐level feature dimension. For example, adaptation across stimulus repetitions has been found for objects with shared visual features (Sayres & Grill‐Spector, 2006), objects with shared category membership (Weiner, Sayres, Vinberg, & Grill‐Spector, 2010), objects with similar dynamic movements (Pyles & Grossman, 2009), or scenes from the same location (Epstein & Morgan, 2012).

**Figure 2:**
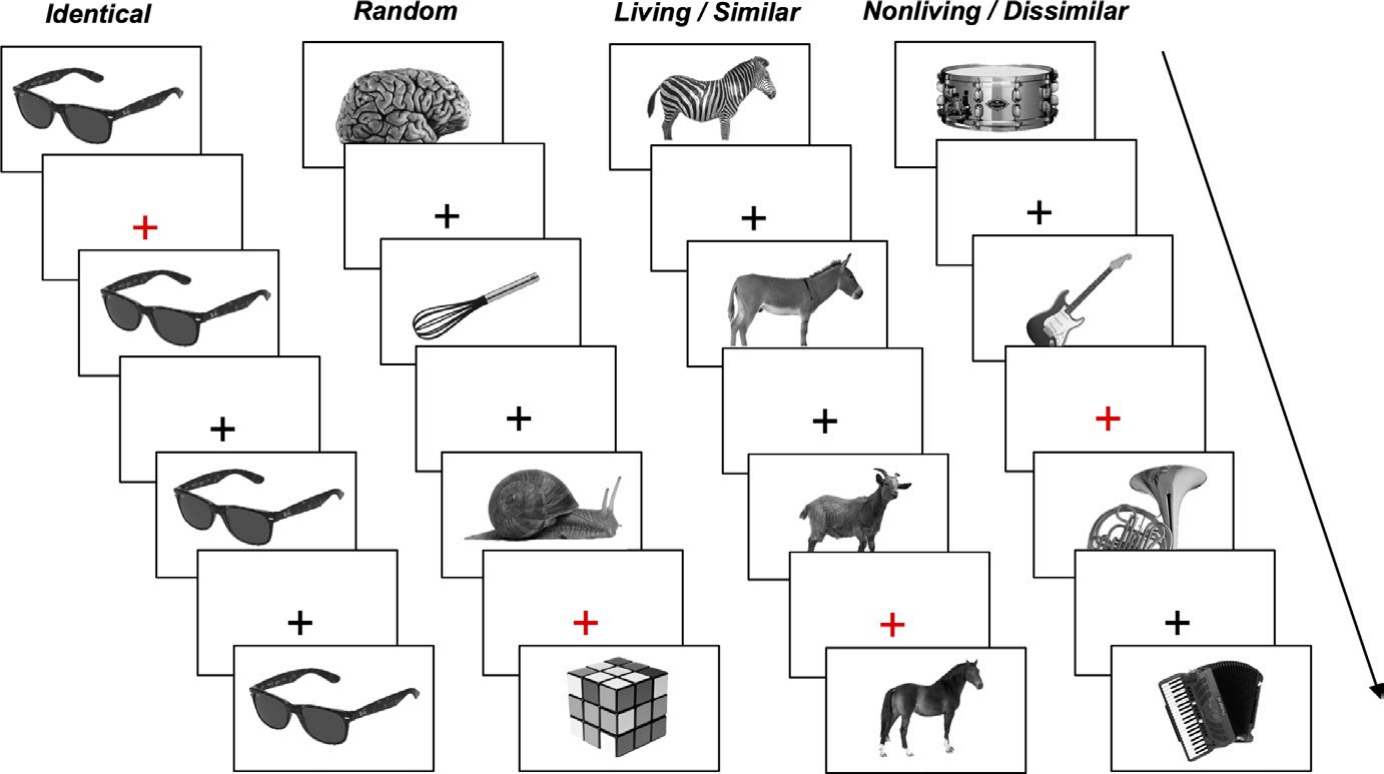
Examples of the block conditions and stimulus presentation order in the fMRI paradigm

In our study, stimulus blocks were composed of groups of 10 objects. There were six different block conditions: identical blocks of the same object image viewed 10 times (expected to elicit maximum ventral visual cortex adaptation), random blocks of 10 distinct and unrelated objects (expected to elicit minimum ventral visual cortex adaptation), and blocks of categorically related object groups (Figure 2). These groups were divided into four conditions: living/similar, nonliving/similar, living/dissimilar, and nonliving/dissimilar. The order of the objects presented within each block and the order of the blocks themselves were randomized for each participant, each object was only viewed once per scan, and the same condition was never viewed twice in a row.

Stimuli were presented in MATLAB (MathWorks, Inc.) using the Psychophysics Toolbox (Brainard, 1997) on an MR‐compatible LCD display (BOLDscreen dimensions: 51.8 × 32.3 cm, resolution: 1,920 × 1,200 pixels) controlled by a MacBook Pro laptop. All of the stimulus images were displayed at the center of the screen in grayscale against a light gray background (20% gray). The images were presented in grayscale to eliminate color‐based perceptual processing and increase focus on form‐based object representations. The images were 400 × 400 pixels (subtending 4.9° × 4.9° of visual angle). Stimuli were adjusted to fit in the 400‐pixel square and not scaled for real‐world size differences between objects.

Each stimulus image was presented for 850 ms with a 350 ms fixation cross between each image presentation, for a total block length of 1,200 ms. Blocks were interleaved with an 8,000 ms passive fixation period to allow the signal to return to baseline before the next block began. Within each block, two or three of the fixation crosses were red (randomized number and order), and the remainder of the crosses were black. Participants were instructed to press the right index finger button of the MR‐compatible button glove when they detected a red fixation cross.

Each experimental scanning session consisted of four runs of the fMRI‐adaptation task, two localizer runs, and high‐resolution anatomical image acquisition. The fMRI‐adaptation task runs each contained 16 blocks (four identical, four random blocks, and two of each of the categorical object blocks), and the initial and final 1,200 ms of each run was a passive fixation period. Participants also completed two runs of the objects versus scrambled object functional localizer runs to identify LOC as a region of interest (ROI) (Grill‐Spector, 2003). The order of each scanning session was as follows: three fMRI‐adaptation task runs, anatomical acquisition, one fMRI‐adaptation run, and two localizer runs.

#### 2.3.2 MRI acquisition

Neuroimaging data were acquired with a 3‐Tesla Siemens Verio scanner equipped with a 32‐channel phased array headcoil located at the Carnegie Mellon University Scientific Imaging & Brain Research Center. High‐resolution anatomical images were acquired for each participant and were used for the coregistration of the functional data (T1‐weighted MPRAGE, 1 mm isovoxel, TR = 2,300 ms, TE = 1.97 ms, flip angle = 9°). Each experiment included two types of functional scans: fMRI‐adaptation scans and localizer scans that were used to identify LOC as an ROI (T2‐weighted gradient EPI, anterior to posterior phase encoding, 3 mm isovoxel, TR = 2,000 ms, TE = 29 ms, flip angle = 79°, 36 axial slices, GRAPPA factor = 2).

#### 2.3.3 fMRI analysis

All neuroimaging data were preprocessed using BrainVoyager (v2.6, Brain Innovations, Inc.). Preprocessing steps included slice‐timing correction, motion correction, and linear trend removal with a temporal high‐pass filter (two cycles per scan). Functional data were manually coregistered to the individual participant's high‐resolution anatomical images.

Lateral occipital cortex was established as an ROI for each participant using the results of the functional localizer runs. LOC was identified as the region of ventral temporal cortex showing more activation for objects relative to scrambled objects using a boxcar model convolved with a hemodynamic response function contrasted using a general linear model (GLM; FDR < 0.01). To assess adaptation effects within this ROI, an event‐related average (ERA) was calculated for all voxels within the ROI, and the timecourse of the ERA was compared separately for each condition. Early visual cortex (EVC), located posteriorly along the calcarine sulcus, was anatomically selected as an exploratory ROI (FDR < 0.05), given its established pattern of adaptation in response to purely visual information (Gardner et al., 2005). Adaptation effects within EVC were also assessed using an ERA analysis to compare timecourse as a function of block condition.

A whole‐brain analysis investigated the wider network of regions that were predicted to show differences in adaptation across both visual and semantic feature processing. GLM contrasts were run between the four semantically related conditions and the random condition (i.e., living/similar, living/dissimilar, nonliving/similar, and nonliving/dissimilar conditions collapsed and contrasted with the random control condition) to establish those regions where the adaptation effect was larger for objects with shared category associations (irrespective of the degree of visual similarity). These regions were thresholded at a *t*‐value greater than two (*p* < .05). Significant activation was found in the angular gyrus (AnG), the precentral gyrus (PrG), and the dorsomedial portion of the prefrontal cortex (DMPFC). Adaptation within these regions was assessed with the ERA timecourse, and the average percent signal change within a block was calculated for each region.

**Figure 3:**
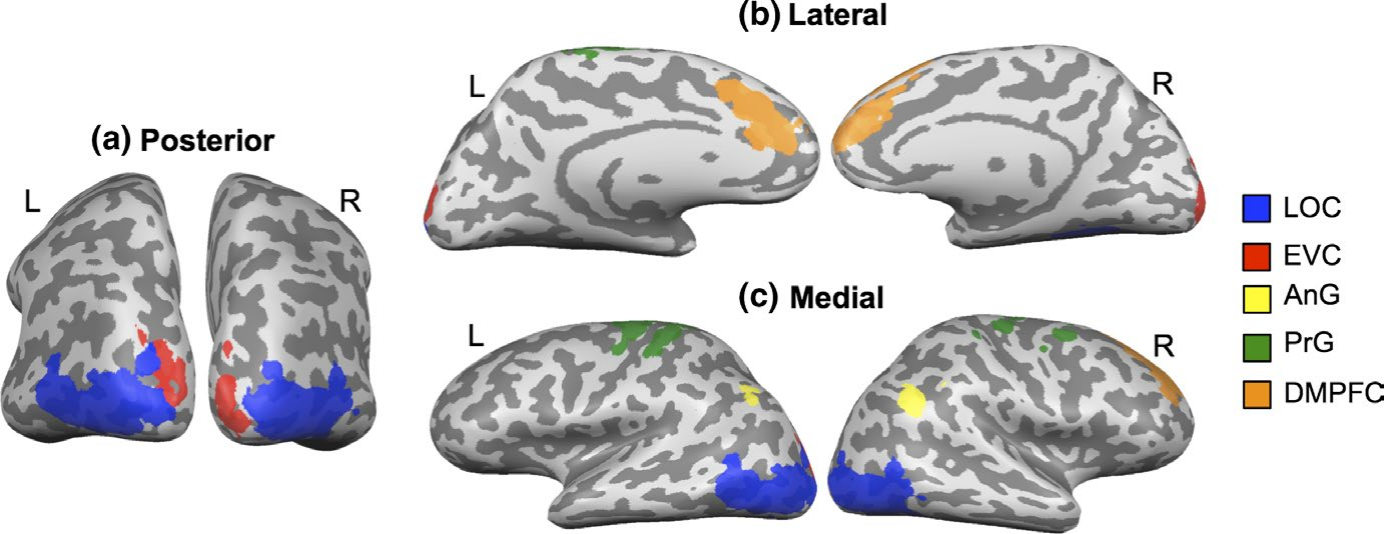
Regions included in the fMRI analysis: lateral occipital cortex (LOC) and early visual cortex (EVC) and semantic regions of activation in angular gyrus (AnG), precentral gyrus (PrG), and dorsomedial prefrontal cortex (DMPFC). Location of the regions is depicted on the inflated cortical surface of one representative participant. (a) Posterior view. (b) Lateral view. (c) Medial view

The ROI and whole‐brain analyses were run separately for the right and left hemispheres, and adaptation across regions did not differ as a function of hemisphere. Therefore, results explicating adaptation effects in the a priori visual cortex ROIs (EVC and LOC) and the wider whole‐brain network (AnG, PrG, and DMPFC) are presented bilaterally. Figure 3 illustrates the locations of these regions.

Segmentation was performed for cortex‐based alignment (Goebel, Esposito, & Formisano, 2006) and group analysis. Gray matter was manually segmented from white matter for each participant. White matter meshes were then smoothed and inflated. To provide the most accurate alignment of cortical surfaces across participants, a cortex‐based alignment approach was used where white matter meshes were first inflated and morphed onto a standardized sphere for all participants, and, subsequently, patterns of gyri and sulci were aligned between participants to create a group reference coordinate space.

## 3 RESULTS

### 3.1 ROI‐based analysis

Adaptation was calculated for each ROI by averaging the overall BOLD percent signal change within a block relative to baseline as a function of condition. Baseline was calculated as the average signal during the three TRs before the block onset. The within‐block calculation was lagged by two TRs to account for the delay of the hemodynamic response. Within‐block percent signal change was then compared across conditions with paired‐samples *t* tests.

**Figure 4:**
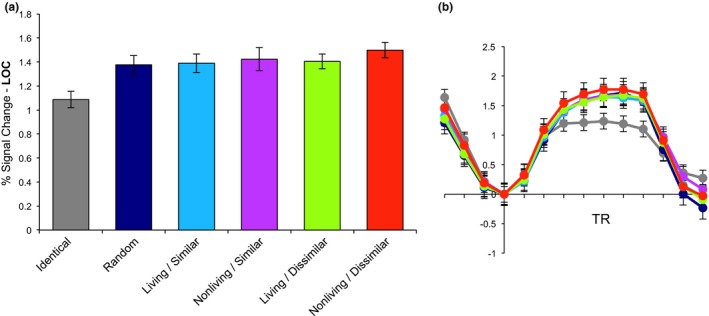
fMRI‐adaptation results (mean ± standard error of the mean) for lateral occipital cortex (LOC). (a) Percent signal change within a block as a function of block condition. (b) ERA timecourses for each condition. The zero point marks the block onset; the plot extends for three TRs beyond the end of the block to show the return to baseline

Within LOC, blocks of identical objects elicited more adaptation than all other conditions (*p* < .001 for all contrasts; Figure 4). There was no main effect of visual similarity, with visually similar objects eliciting the same level of adaptation as visually dissimilar objects within LOC (*t*(19) = −1.15, *p* = .26). There was also no main effect of living/nonliving category membership within LOC (*t*(19) = −1.73, *p* = .10). Among the four categorically related conditions, a repeated‐measures ANOVA did not reveal a significant interaction between visual similarity and living/nonliving category membership (*F*(3,19) = 1.73, *p* = .17).

**Figure 5:**
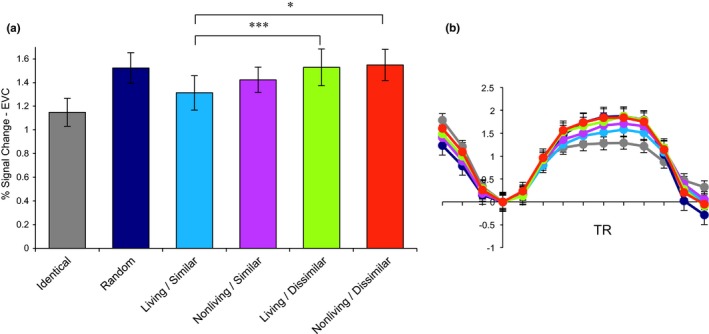
fMRI‐adaptation results (mean ± standard error of the mean) for early visual cortex (EVC). (a) Percent signal change within a block as a function of block condition. (b) ERA timecourses for each condition. The zero point marks the block onset; the plot extends for three TRs beyond the end of the block to show the return to baseline. *p < .05; ***p < .005

Within EVC, blocks of identical objects elicited more adaptation than all other conditions (*p* < .05 for all contrasts; Figure 5). Random objects adapted less overall than visually similar objects (*p *< .05), but there was no difference in adaptation effects between random and visually dissimilar objects within EVC (*p* = .77). There was a main effect of visual similarity within EVC, with visually similar objects having significantly greater adaptation as compared to visually dissimilar objects (*t*(19) = −3.23, *p* < .005). The main effect of living/nonliving category membership was not significant when collapsed across visual similarity (*t*(19) = −0.96, *p* = .35). Among the four categorically related conditions, a repeated‐measures ANOVA revealed a significant interaction between visual similarity and living/nonliving category membership (*F*(3,19) = 3.73, *p* < .05). Post hoc analyses indicated the largest adaptation effect in the living/similar condition, with these objects showing a significantly smaller percent signal change as compared to both the living/dissimilar condition (*t*(19) = −3.38, *p* < .005) and the nonliving/dissimilar condition (*t*(19) = −2.78, *p* < .05).

### 3.2 Whole‐brain analysis

**Figure 6:**
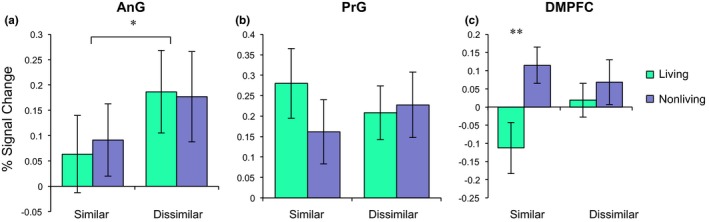
fMRI‐adaptation results (mean ± standard error of the mean) for the semantic network. Plots show percent signal change within a block as a function of block condition in (a) angular gyrus (AnG), (b) precentral gyrus (PrG), and (c) dorsomedial prefrontal cortex (DMPFC). *p < .05; **p < .01

Within‐block adaptation was calculated for each region by averaging the overall percent signal change within a block as a function of condition, lagged by two TRs to account for the delay of the hemodynamic response. Since the selection of brain regions showing adaptation was based on a contrast with the random condition, the random condition is dropped from the present results. The identical condition is also excluded from these analyses, as this analysis was designed to only consider different patterns in adaptation between the four categorical conditions (Figure 6).

Within AnG, there was a main effect of visual similarity within AnG, with visually similar objects having greater adaptation as compared to visually dissimilar objects (*t*(19) = −2.37, *p* < .05). However, there was no main effect of adaptation for living versus nonliving category membership (*t*(19) = −0.13, *p* = .90). The interaction of visual similarity and living/nonliving category membership was not significant (*F*(3,19) = 1.26, *p* = .30).

Within PrG, there was no main effect of visual similarity (*t*(19) = 0.04, *p* = .97), no main effect of living versus nonliving category membership in the PrG (*t*(19) = 0.91, *p* = .38). The interaction of visual similarity and living/nonliving category membership was also not significant (*F*(3,19) = 0.71, *p* = .55).

Within DMPFC, the main effect of living/nonliving category membership was significant, with living objects showing greater adaptation as compared to nonliving objects (*t*(19) = −2.10, *p* < .05). There was no main effect of visual similarity (*t*(19) = −0.75, *p* = .46). There was a significant interaction between visual similarity and living/nonliving category membership (*F*(3,19) = 3.26, *p* < .05). Post hoc analyses indicated that the largest adaptation effect was observed between the living and nonliving visually similar conditions, but with living objects adapting significantly more (*t*(19) = −2.92, *p* < .01). There was no difference in adaptation between the living and nonliving visually dissimilar categories (*t*(19) = −0.69, *p* = .50). The greater difference in adaptation within the visually similar condition indicates that both visual and semantic features may contribute to the maintenance of object category boundaries within DMPFC.

## 4 DISCUSSION

The principle finding of our study is that shared visual features appear to contribute to category‐selective responses within a distributed, whole‐brain object processing network. Within the visual cortex, we observed expected effects of visual similarity in EVC, but we did not observe differential sensitivity to visual or semantic features within the LOC (part of the ventral visual stream). Looking more broadly, an extensive network of regions associated with object category processing, including AnG, PrG, and DMPFC, were found to adapt more in response to shared semantic category membership (i.e., living vs. nonliving compared with random), irrespective of the degree of visual similarity. However, visual similarity also contributed to category representations within these nonvisual regions. AnG showed increased adaptation for visually and semantically similar objects, while the DMPFC had different patterns of adaptation for living versus nonliving object categories, with the most adaptation for living objects that were also visually similar, indicating integration of both forms of information within this frontal processing region. Overall, our findings support a neural architecture for category representation that is distributed across a network of brain regions sensitive to differing combinations of visual and semantic features.

We predicted that LOC would be sensitive to perceptual feature overlap and exhibit increased neural adaptation for visually similar relative to visually dissimilar object categories. However, we did not observe this effect, with this critical object processing region of the ventral visual cortex instead demonstrating no significant difference in its adaptation responses across these conditions. However, we did observe that identical objects gave rise to the largest adaptation effects in LOC—unsurprising in that LOC is typically associated with the processing of object form (Grill‐Spector, Kourtzi, & Kanwisher, 2001). However, the unexpected finding of no significant category differences in LOC suggests that this region may not be differentially sensitive to visual versus semantic features. Rather, the LOC may play a role in object identification and/or generalization across categories (Grill‐Spector et al., 2001; Grill‐Spector & Weiner, 2014), as opposed to maintaining divisions between category boundaries (Eger, Ashburner, Haynes, Dolan, & Rees, 2008). As such, the LOC may encode higher‐order object properties, including both visual form and semantic category membership and, consequently, may not differentiate between the unique elements that are bound to both perceptual and semantic object identity (e.g., *tables* have *legs*, and *birds* have *beaks*). In the context of object recognition, this kind of information (as opposed to collapsing across shared features) may be more effective for identifying individual objects within and across categories.

Additional evidence for a lack of a clear distinction between visual and semantic categorization in ventral visual cortex comes from neuroimaging of the visual processes supporting both basic and subordinate‐level recognition across both perceptual and conceptual tasks (Gauthier, Anderson, Tarr, Skudlarski, & Gore, 1997). Consistent with previous findings indicating that visual regions encode features related to object category, both perceptual and conceptual tasks recruited parts of the fusiform and inferior temporal gyri within the ventral visual cortex (Gauthier et al., 1997; see also Gauthier et al., 2000). Gauthier, Curran, Curby, and Collins (2003) manipulated perceptual and semantic category knowledge more directly by using novel objects to examine the organization of categories without the confound of known object names or prior conceptual knowledge of the test categories. Novel objects that were assigned to the same novel semantic category were subsequently perceived as being more visually similar. This finding demonstrates knowledge of shared conceptual category membership can influence high‐level visual representations, a point that is consistent with our finding of no differential adaptation effect for visually similar versus visually dissimilar categories in LOC.

We should note that the pattern of responses we observed in the LOC differ from those reported in previous research. In particular, earlier studies have found that the ventral visual cortex appears to be organized into category‐selective regions that respond preferentially to a particular object category or categories (e.g., Bi et al., 2016; Caramazza & Mahon, 2003; Hutchison, Culham, Everling, Flanagan, & Gallivan, 2014; Peelen & Downing, 2017). However, the finding of category selectivity in visual areas does not, in and of itself, establish that semantics play any role in this level of organization. Instead, our results suggest that those visual processes characterized as “category‐selective” may arise solely due to the differential representation of individual object features that ultimately help to define object categories (e.g., Cutzu & Tarr, 1997; Grill‐Spector, 2003; Lee & Baker, 2016; O'Reilly et al., 2013; Quinn, Eimas, & Tarr, 2001; Rice, Watson, Hartley, & Andrews, 2014).

The overall pattern of adaptation within EVC was consistent with our predictions. In particular, we observed a higher level of adaptation for visually similar objects relative to visually dissimilar objects. Unexpectedly, we also observed greater adaptation for living objects relative to nonliving objects within EVC. This latter finding hints that some aspects of higher‐order conceptual knowledge may be reflected in earlier visual processing or that there exist relatively low‐level perceptual differences sufficient to separate these two categories. However, supporting the former possibility, we note that EVC receives feedback from higher‐order visual processing regions (e.g., Gilbert & Sigman, 2007) and motor planning regions (Gutteling et al., 2015). One possibility is that the higher adaptation level we observed for living things in EVC is grounded in the category‐relevant informativeness of perceptual and conceptual features processed in more anterior brain regions.

Our results also explicate some of the possible roles for different regions within a distributed, whole‐brain network associated with the representation of object categories. First, we unexpectedly observed adaptation in AnG, a region that is held to be an association area that integrates information across multiple stimulus modalities (Bonnici, Richter, Yazar, & Simons, 2016; Ramanan, Piguet, & Irish, 2017). The sensitivity of AnG to both visual and semantic features suggests that this region may be influenced by distinct brain regions that process perceptual and conceptual knowledge. As such, AnG may be an early point in the hierarchy of object category integration, mechanistically passing visual information forward to higher‐order conceptual processing regions (Binder, Desai, Graves, & Conant, 2009). Supporting this view, Diaz & McCarthy (2007) reported that AnG responds in a consistent manner across a range of word categories, with similar responses for content words (e.g., *bear*, *hat*, and *ship*) as compared to function words that have a lexical meaning but that have low conceptual complexity (e.g., *circa*, *nowhere*, and *thine*). The consistent response we observed for living and nonliving objects, paired with the larger adaptation effect we observed for visually similar objects, reinforces the idea that AnG may be less sensitive than previously thought to higher‐order conceptual knowledge.

Consistent with the overall view of a neural object processing hierarchy in the brain, we observed that moving in an anterior direction reveals a shift from perceptually based adaptation to semantically based adaptation. In particular, PrG was identified as a conceptual processing region in our whole‐brain analysis, as it adapted more in response to objects that shared living or nonliving category membership compared to random objects with no conceptual association. This adaptation effect is in accord with previous literature; PrG has been shown to be involved in the integration of object identity and its associated actions, with increased responses in PrG when an object is viewed in the context of being used (Liljeström et al., 2008; Thioux & Keysers, 2015). However, although PrG has been construed as a conceptual area, we did not observe the predicted main effect of living versus nonliving category membership within this region.

Finally, we observed semantic category adaptation in DMPFC, with greater adaptation for living then nonliving objects. Note that the largest adaptation effect was seen for the living/similar category of objects, indicating that information about visual similarity may also be projected to this frontal, conceptual processing region. DMPFC is adjacent to regions important for attention, such as anterior cingulate cortex, leading some researchers to suggest that DMPFC may play a role in shorter‐term sustained semantic adaptation across the duration of a block (Binder et al., 2009). Our finding of category‐based adaptation in DMPFC differs from previous research that has alternatively linked the ventrolateral prefrontal cortex (VLPFC) with semantic retrieval and top‐down control of longer‐term memory representations (Martin & Chao, 2001; Nozari & Thompson‐Schill, 2016; Thompson‐Schill, 2003). However, the blocked design used in our experiment does not require long‐term maintenance or top‐down control as may be recruited by the more memory‐based tasks used in these earlier studies.

Taken together, the network of visual and semantic processing regions explicated in our present study point to the importance of more frontal brain regions in maintaining knowledge‐based representations of semantic features, while also demonstrating the influence of visual similarity in defining category boundaries. This organization is consistent with a large body of literature that closely links to the sensory/functional theory of object processing (e.g., Warrington & McCarthy, 1987). Our results indicating an interaction of visual and semantic features in the EVC and DMPFC are also consistent with the idea that living things are more commonly classified based on their shared visual features, while nonliving things are more commonly classified based on their shared functional properties (e.g., Thompson‐Schill, 2003).

An alternative way of framing this division of labor is in terms of the complementary functional roles for these distinct object processing regions. More specifically, sensory‐associated regions in posterior portions of the brain may be crucial for individual feature extraction, while memory‐associated frontal regions in more anterior portions of the brain may be linked to semantic knowledge and category boundaries (Binder et al., 2009). This characterization is consistent with our observation that AnG shows greater adaptation for visually similar objects with shared features regardless of the living/nonliving distinction. In contrast, DMPFC had differential adaptation effects for the living versus nonliving distinction, a finding that places semantic category boundaries in these frontal regions.

While there were no significant differences in Gabor distance between living and nonliving categories (collapsed across visual similarity), it is important to note that the observed adaptation effects for the living/similar condition in particular may be driven by the fact that stimuli in this condition have the highest degree of pixel overlap (i.e., least Gabor distance), which is a limitation of the current study design. We cannot rule out the influence of low‐level visual features in the processing of stimuli in this category and may only conclude that adaptation in response to living/similar objects represents combined processing of visual and semantic features. Another limitation of our present study was that the network of brain regions sensitive to both visual and semantic similarity was identified by collapsing across the four semantically associated conditions. Given this design, we are able to identify the relative contributions of both visual and semantic features across the brain, but we were not able to draw any binary distinctions between purely visual versus purely semantic processing regions. In particular, increased adaptation for living/dissimilar objects relative to nonliving/dissimilar objects provides some evidence in support of a category‐selective representation, given that these object categories did not significantly differ in their degree of shared visual features or pixel overlap. These methodological details do not detract from our main finding, which is that visual similarity was found to influence category representations in frontal brain regions typically considered to be “nonvisual” (e.g., DMPFC).

In summary, our results suggest that a distributed network of processing regions is responsible for the integration of a wide range of object features. Greater neural adaptation for visually similar objects within a category relative to their dissimilar counterparts throughout a distributed network suggests that visual features influence category‐selective processing in nonvisual regions. In particular, we observed that perceptual feature overlap between objects modulated responses in anteriorly located processing regions, including premotor and prefrontal cortices. Overall, we posit that 1) this network for category representation reflects distinct brain regions that efficiently extract the most relevant features for category membership; and 2) the transfer of visual information among these regions is fundamental to the neural representation of object categories.

## CONFLICT OF INTEREST

MJT is a cofounder of Neon Laboratories and holds the following patent: “Automated thumbnail selection for online video” (WO 2014078530). LWV and JAP have no conflicts to declare.

## Data Availability

The data that support the findings of this study are available from the corresponding author upon reasonable request.
